# Expert consensus on the diagnosis and treatment of cemental tear

**DOI:** 10.1038/s41368-025-00381-9

**Published:** 2025-08-22

**Authors:** Ye Liang, Hongrui Liu, Chengjia Xie, Yang Yu, Jinlong Shao, Chunxu Lv, Wenyan Kang, Fuhua Yan, Yaping Pan, Faming Chen, Yan Xu, Zuomin Wang, Yao Sun, Ang Li, Lili Chen, Qingxian Luan, Chuanjiang Zhao, Zhengguo Cao, Yi Liu, Jiang Sun, Zhongchen Song, Lei Zhao, Li Lin, Peihui Ding, Weilian Sun, Jun Wang, Jiang Lin, Guangxun Zhu, Qi Zhang, Lijun Luo, Jiayin Deng, Yihuai Pan, Jin Zhao, Aimei Song, Hongmei Guo, Jin Zhang, Pingping Cui, Song Ge, Rui Zhang, Xiuyun Ren, Shengbin Huang, Xi Wei, Lihong Qiu, Jing Deng, Keqing Pan, Dandan Ma, Hongyu Zhao, Dong Chen, Liangjun Zhong, Gang Ding, Wu Chen, Quanchen Xu, Xiaoyu Sun, Lingqian Du, Ling Li, Yijia Wang, Xiaoyuan Li, Qiang Chen, Hui Wang, Zheng Zhang, Mengmeng Liu, Chengfei Zhang, Xuedong Zhou, Shaohua Ge

**Affiliations:** 1 Department of Periodontology, School and Hospital of Stomatology, Cheeloo College of Medicine, Shandong University & Shandong Key Laboratory of Oral Tissue Regeneration & Shandong Engineering Research Center of Dental Materials and Oral Tissue Regeneration & Shandong Provincial Clinical Research Center for Oral Diseases, Jinan, China; 2 Department of Periodontology, Nanjing Stomatological Hospital, Affiliated Hospital of Medical School, Institute of Stomatology, Nanjing University, Nanjing, China; 3 Department of Periodontics, School and Hospital of Stomatology, China Medical University, Shenyang, China; 4 State Key Laboratory of Oral & Maxillofacial Reconstruction and Regeneration, National Clinical Research Center for Oral Diseases, Shaanxi International Joint Research Center for Oral Diseases, Department of Periodontology, School of Stomatology, The Fourth Military Medical University, Xi’an, China; 5 Department of Periodontology, The Affiliated Stomatological Hospital of Nanjing Medical University, Jiangsu Key Laboratory of Oral Diseases, Nanjing Medical University, Nanjing, China; 6 Department of Stomatology, Beijing Chaoyang Hospital, Capital Medical University, Beijing, China; 7 Department of Implantology, Stomatological Hospital and Dental School of Tongji University, Shanghai Engineering Research Center of Tooth Restoration and Regeneration, Shanghai, China; 8 Key Laboratory of Shaanxi Province for Craniofacial Precision Medicine Research, College of Stomatology, Xi’an Jiaotong University, Xi’an, China; 9 Department of Oral Medicine, The Second Affiliated Hospital, School of Medicine, Zhejiang University, Hangzhou, China; 10 Department of Periodontology, Peking University School and Hospital of Stomatology & National Center of Stomatology & National Clinical Research Center for Oral Diseases & National Engineering Laboratory for Digital and Material Technology of Stomatology & Beijing Key Laboratory of Digital Stomatology & Research Center of Engineering, Beijing, China; 11 Hospital of Stomatology, Guangdong Provincial Key Laboratory of Stomatology, Guanghua School of Stomatology, Sun Yat-sen University, Guangzhou, China; 12 State Key Laboratory of Oral & Maxillofacial Reconstruction and Regeneration, Key Laboratory of Oral Biomedicine Ministry of Education, Hubei Key Laboratory of Stomatology, School & Hospital of Stomatology, Wuhan University; Department of Periodontology, School & Hospital of Stomatology, Wuhan University, Wuhan, China; 13 Laboratory of Tissue Regeneration and Immunology and Department of Periodontics, Beijing Key Laboratory of Tooth Regeneration and Function Reconstruction, School of Stomatology, Capital Medical University, Beijing, China; 14 Dalian Stomatological Hospital, Dalian, China; 15 Department of Periodontology, Shanghai Ninth People’s Hospital, Shanghai Jiao Tong University School of Medicine; College of Stomatology, Shanghai Jiao Tong University; National Center for Stomatology; National Clinical Research Center for Oral Diseases; Shanghai Key Laboratory of Stomatology; Shanghai Research Institute of Stomatology, Shanghai, China; 16 State Key Laboratory of Oral Diseases & National Center for Stomatology & National Clinical Research Center for Oral Diseases & Department of Periodontics, West China Hospital of Stomatology, Sichuan University, Chengdu, China; 17 Stomatology Hospital, School of Stomatology, Zhejiang University School of Medicine, Zhejiang Provincial Clinical Research Center for Oral Diseases, Key Laboratory of Oral Biomedical Research of Zhejiang Province, Cancer Center of Zhejiang University, Hangzhou, China; 18 Department of Stomatology, Beijing TongRen Hospital, Capital Medical University, Beijing, China; 19 Department of Stomatology, Tongji Hospital, Tongji Medical College, Huazhong University of Science and Technology, Wuhan, China; 20 Department of Endodontics, Stomatological Hospital and Dental School of Tongji University, Shanghai Engineering Research Center of Tooth Restoration and Regeneration, Shanghai, China; 21 Department of Periodontics, Stomatological Hospital and Dental School of Tongji University, Shanghai Engineering Research Center of Tooth Restoration and Regeneration, Shanghai, China; 22 School and Hospital of Stomatology, Tianjin Medical University, Tianjin, China; 23 Department of Endodontics, School and Hospital of Stomatology, Wenzhou Medical University, Wenzhou, China; 24 Department of Endodontics, First Affiliated Hospital of Xinjiang Medical University, and College of Stomatology of Xinjiang Medical University, Urumqi, China; 25 Department of Endodontics, School and Hospital of Stomatology, Cheeloo College of Medicine, Shandong University & Shandong Provincial Key Laboratory of Oral Tissue Regeneration & Shandong Provincial Engineering Laboratory for Dental Materials and Oral Tissue Regeneration & Shandong Provincial Clinical Research Center for Oral Diseases, Jinan, China; 26 School and Hospital of Stomatology, Zunyi Medical University, Zunyi, China; 27 Shanxi Medical University School and Hospital of Stomatology, Shanxi Province Key Laboratory of Oral Diseases Prevention and New Materials, Taiyuan, China; 28 Department of Prosthodontics, School and Hospital of Stomatology, Wenzhou Medical University, Wenzhou, China; 29 Department of Operative Dentistry and Endodontics, Hospital of Stomatology, Guanghua School of Stomatology, Sun Yat-Sen University & Guangdong Provincial Key Laboratory of Stomatology, Guangzhou, China; 30 Department of Endodontics, School and Hospital of Stomatology, China Medical University; Liaoning Provincial Key Laboratory of Oral Diseases, Shenyang, China; 31 Department of Stomatology, The Affiliated Hospital of Qingdao University, Qingdao, China; 32 Department of Endodontics, Stomatological Hospital, School of Stomatology, Southern Medical University, Guangzhou, China; 33 Department of Stomatology, The First Affiliated Hospital of Zhengzhou University, Zhengzhou, China; 34 Stomatology Center, Affiliated Hospital of Hangzhou Normal University, Hangzhou, China; 35 School of Stomatology, Hangzhou Normal University, Hangzhou, China; 36 School of Stomatology, Shandong Second Medical University, Weifang, China; 37 Key Laboratory of Oral Diseases Research of Anhui Province, College & Hospital of Stomatology, Anhui Medical University, Hefei, China; 38 The Second Hospital of Shandong University, Jinan, China; 39 Department of Periodontology, Linyi People’s Hospital, Shandong Second Medical University, Linyi, China; 40 Department of Stomatology, The Fifth People’s Hospital of Jinan, Jinan, China; 41 Suzhou Stomatological Hospital, Suzhou, China; 42 Tianjin Stomatological Hospital, School of Medicine, Nankai university, Tianjin, China; 43 Department of Stomatology, Qilu Hospital of Shandong University (Qingdao), Cheeloo College of Medicine, Shandong University, Qingdao, China; 44 Restorative Dental Sciences, Faculty of Dentistry, The University of Hong Kong, Hong Kong, China; 45 State Key Laboratory of Oral Diseases & National Center for Stomatology & National Clinical Research Center for Oral Diseases & Department of Cariology and Endodontics, West China Hospital of Stomatology, Sichuan University, Chengdu, China

**Keywords:** Oral diseases, Diagnosis

## Abstract

Cemental tear is a rare and indetectable condition unless obvious clinical signs present with the involvement of surrounding periodontal and periapical tissues. Due to its clinical manifestations similar to common dental issues, such as vertical root fracture, primary endodontic diseases, and periodontal diseases, as well as the low awareness of cemental tear for clinicians, misdiagnosis often occurs. The critical principle for cemental tear treatment is to remove torn fragments, and overlooking fragments leads to futile therapy, which could deteriorate the conditions of the affected teeth. Therefore, accurate diagnosis and subsequent appropriate interventions are vital for managing cemental tear. Novel diagnostic tools, including cone-beam computed tomography (CBCT), microscopes, and enamel matrix derivatives, have improved early detection and management, enhancing tooth retention. The implementation of standardized diagnostic criteria and treatment protocols, combined with improved clinical awareness among dental professionals, serves to mitigate risks of diagnostic errors and suboptimal therapeutic interventions. This expert consensus reviewed the epidemiology, pathogenesis, potential predisposing factors, clinical manifestations, diagnosis, differential diagnosis, treatment, and prognosis of cemental tear, aiming to provide a clinical guideline and facilitate clinicians to have a better understanding of cemental tear.

## Introduction

Cemental tear can be an incomplete or complete detachment of the cementum along the cemento-dentinal junction (CDJ) or a partial detachment along the incremental lines within the body of the cementum.^1–3^ It is a specific type of root surface fracture involving cementum; sometimes, the tear also involves dentin, termed cemento-dentinal tear.^4^ There are no specific clinical symptoms at the early stage of cemental tear forming; surrounding tissue is gradually destroyed with progression resulting from the low-grade inflammation induced by mechanical stimulation of torn fragments,^5^ leading to sinus tract, isolated deep periodontal pocket, purulent discharge, occlusal pain, etc. The diagnosis of cemental tear is challenging because of its clinical presentations similar to vertical root fracture (VRF), periodontitis, and apical periodontitis.^4,6,7^ In addition, low awareness of cemental tears can also lead to misdiagnosis and unnecessary treatment, even for experienced clinicians, posing significant clinical dilemmas.^3,8^ Therefore, a clinical guideline is needed for improving cemental tear management, and this consensus aims to summarize recent progress on the epidemiology, pathogenesis, possible predisposing factors, clinical presentation, diagnosis, differential diagnosis, treatment, and treatment outcomes of cemental tear and to help clinicians enhance awareness of cemental tear and develop feasible treatment plans.

## Epidemiology of cemental tear

Most literature on cemental tear consists of case reports and reviews, and epidemiological analysis is limited. Currently, only two articles based on radiographic images have reported the prevalence of cemental tear.^9,10^ Keskin et al. conducted a retrospective observational study based on periapical radiographs of 4 629 permanent teeth of 1 451 adult patients in Turkey reporting a prevalence of 0.89%,^9^ while Özkan et al. reported a prevalence of 1.9% by assessment of 813 cone-beam computed tomography (CBCT) images in another region of Turkey.^10^ More comprehensive information with a three-dimensional (3D) perspective in CBCT images which can facilitate detecting cemental tear compared with periapical images, as well as the different study populations and sample sizes, may result in the different prevalence in the above two studies. However, the diagnosis of cemental tear in both studies was verified by radiography alone and lack of clinical examination,^3^ and further studies with larger sample sizes in different regions should be conducted to understand the prevalence of cemental tear comprehensively. Moreover, comprehensive clinical information should be included in further studies to better investigate the epidemiological characteristics of cemental tear in different populations, such as in a healthy population or a population with periodontal diseases. There was no report on the incidence of cemental tear till now; perhaps its rare occurrence in clinics and low awareness of this disease hinder the data collection.^3^

## Histology of cementum

Firstly, knowing the histology of the cementum would facilitate the understanding of the underlying mechanisms of cemental tear formation. Cementum is the mineralized tissue similar to bone covering the entire root surface, contributing to periodontal support along with periodontal ligament and alveolar bone.^11,12^ Unlike bone, cementum has no blood vessels, Haversian canals, or nerves, and it is resistant to normal remodeling and resorption. Cementum is thinner near the cervical part of the root, about 20 to 50 µm thick, and about 150 to 200 µm thick at the root apex,^13^ and its thickness gradually increases with age due to the lack of dynamic remodeling.^11,14^ Cementum can be histologically classified into cellular and acellular cementum by the inclusion or absence of cementocytes. Generally, acellular cementum is thinner and covers the cervical part of the root, while cellular cementum is thicker and covers the apical part of the root.^11^ Additionally, cementum contains two types of fibers: extrinsic and intrinsic. Extrinsic fibers are secreted by fibroblasts and embedded in the periodontal ligament, and intrinsic fibers are laid down only by cementoblasts and located within the cementum.^11^ Based on the existence of cementocytes and the different structures of cementum, Schroeder proposed the current classifications of cementum (Fig. S1).^11,15^

1. **Acellular afibrillar cementum (AAC):** AAC comprises the mineralized matrix without collagen fibers and cementocytes. It is often found as the isolated cementum island or the most cervical part of cementum on the enamel surface coronal to the cemento-enamel junction (CEJ). AAC does not participate in tooth-periodontal ligament attachment because there is no fiber insertion.
2. **Acellular extrinsic fiber cementum (AEFC):** AEFC constitutes the primary cementum (formed before the tooth reaches the occlusal plane), containing radially arranged and densely packed extrinsic fibers without cementocytes. This kind of cementum is a thin layer with a typical thickness of 5-10 µm laid down directly on the root surface, covering 60%–90% of the total root length in single-rooted teeth and cervical 1/3 to 1/2 in multi-rooted teeth. AEFC usually participates in tooth support because the extrinsic fibers inside are connected with the principal fibers in the periodontal ligament. For thick AEFC, incremental lines can be observed, which are highly mineralized and formed in the resting phase during intermittent AEFC formation.^11^ The extrinsic fiber orientation changes at the incremental lines in many cases, which is considered correlated with tooth eruption.^15^ AEFC has less change under extrinsic factors such as mechanical load, with a slower but constant growth rate of approximately 1 µm per year.^16,17^
3. **Cellular intrinsic fiber cementum (CIFC):** CIFC usually exists with cellular mixed stratified cementum (CMSC), which participates in cementum repair.
4. **CMSC:** CMSC is the major component of secondary cementum (formed after the tooth reaches the occlusal plane) and is a thicker layer of cementum commonly found in apical and furcation areas covering primary acellular cementum or the root dentin in the apical region of the root.^18^ CMSC usually consists of stratified CIFC but contains both extrinsic and intrinsic fibers. It can be subdivided into extrinsic fiber-rich CIFC, extrinsic fiber-poor CIFC, and extrinsic fiber-free CIFC based on the density of extrinsic fibers. The thickness of this cellular cementum increases throughout life, and its maximum thickness in middle-aged individuals is between 400 and 600 µm in incisors, about 500 µm in canines, between 300 and 1 000 µm in premolars, and between 700 and 1 500 µm in molars.^15^ Additionally, typical CMSC is stratified by incremental lines because of the resting phase of cementum deposition. The degree of arrangement of intrinsic fibers and mineralization of cementum increases toward the periodontal ligament side between adjacent incremental lines.^11,19,20^ The structural proportion of the lamellae has been interpreted as the gradual recovery of fiber-organizing and mineralization-inducing activity in cementoblasts after the resting phase.^11^

In conclusion, cementum is a complex of collagen fibers and calcified substances connecting dentin and periodontal ligament, except AAC. According to the surrounding environment or requirement (adaptation and tooth anchorage), different types of cementum are generated, i.e. AEFC, CMSC, and CIFC.^11^ As an interconnection system, cementum is continuous with periodontal ligament by extrinsic fibers, and it is attached to dentin via a 100–200 µm thick interface (CDJ) within which a 10–50 µm wide hygroscopic proteoglycan (PG)-rich layer exists. Within the CDJ, collagen fibers from cementum that transverse radially to the dentin are tied with PGs, which differ from the interconnection between cementum and periodontal ligament.^21,22^ Although the microstructure of cementum has been extensively studied since it was first described in the 1830s, further research is still needed to deepen our understanding of cementum histology, particularly regarding the microstructure and deposition processes of various cementum types, the mechanisms of fiber connections at the cementum-periodontal ligament interface, and the age-related or mechanically-induced changes at the CDJ. A comprehensive exploration of these aspects is essential for advancing our knowledge of cemental pathologies and facilitating the development of effective strategies for cementum regeneration.

## Pathogenesis and possible predisposing factors of cemental tear

The etiology of cemental tear is unclear, and two mechanisms have been proposed for the formation and progression of cemental tear based on previous studies, namely internal and external factors.^2,3^ Internal factors are mainly innate and related to the inherent structural weakness of cementum. On the one hand, the connective tissue at CDJ contains many glycoproteins, especially bone sialoprotein and osteopontin,^23^ which contributes to the weaker fibrillar interconnection between cementum and dentin than the connection between cementum and periodontal ligament, as these glycoproteins may disrupt the continuity of fibers connecting different tissues.^23^ On the other hand, the structural weakness of the secondary cementum, consisting of cementocytes in the lacunae and intrinsic collagen fibers, also predisposes it to be detached.^2,21^ External factors are mainly related to repeated stress from occlusal trauma or sudden stress from dental trauma.^6,24–27^ Moreover, several possible predisposing factors will be discussed later.

Once a cemental tear forms, the torn fragment mechanically irritates adjacent periodontal tissues under functional occlusal load, inducing persistent low-grade inflammation.^24^ With the progression of inflammation, alveolar bone defects gradually enlarge, eventually creating periodontal pockets or sinus tracts that communicate directly with the oral cavity. These pathological spaces facilitate plaque accumulation and retention, thus exacerbating periodontal and periapical tissue destruction.^24^ Additionally, microbial pathogens can continuously invade through exposed dentinal tubules, accessory canals, or lateral canals, potentially leading to pulp inflammation.^3^ Notably, when the cemental tear occurs in the apical region without communication to the oral cavity, the periapical inflammation typically remains sterile, unless there is concomitant primary root canal infection.^3^

While the pathogenesis of cemental tear remains incompletely understood, current evidence indicates several possible predisposing factors, which have been summarized in Table S1 to enhance awareness of this disease, including age, gender, systemic condition, tooth type, occlusal trauma, history of periodontitis, and history of dental trauma. Although these factors are theoretically plausible, it must be emphasized that current evidence derives primarily from case reports or retrospective series (Oxford CEBM Level 4 evidence). Their clinical correlations still require validation through clinical studies with strong evidence.

### Age

Cemental tear is highly prevalent in individuals over 60 years old, possibly related to the gradual thickening of cementum with age.^3,7,28^ Throughout a person’s lifespan, the thickness of cementum in the elderly can be 3–5 times greater than in the youth.^4,29,30^ The degree of thickening varies depending on the tooth type and root location, with the most significant thickening occurring at the apex. Additionally, the dominance of collagenous and noncollagenous protein in CDJ with water retention characteristics has been speculated to facilitate dissipation of accumulated function-related stresses. However, the hygroscopic activity of CDJ decreases over time because of mineral formation across the CDJ.^31^ With increasing age, the adhesion strength of glycoproteins at CDJ also decreases, and the collagen that limits the stretching of periodontal ligament fibers weakens.^4^ When the extension of periodontal ligament fibers cannot be properly controlled, it may lead to the detachment of cementum from the dentin surface.^4^

### Gender

Gender is one of the potential predisposing factors for cemental tear. A higher proportion of teeth with cemental tear in males (77.5%) compared with females (22.5%) was reported in Lin’s study, which is a multicenter retrospective study including 71 teeth with cemental tear conducted in Taiwan,^7^ possibly due to greater occlusal forces or differences in cementum structure between genders.^3,4,7^ Lee et al. reviewed 41 cases in the published case reports and revealed a larger percentage (63%) of male patients.^3^ However, there was no significant correlation between gender and occurrence of cemental tear in Keskin’s study or Özkan’s study,^9,10^ which might be due to only a small number of samples detected with cemental tear relative to the total sample size, and more cases need to be collected for further analysis.

### Systemic condition

A special case report of one individual with 14 affected teeth revealed that certain systemic conditions could be potential predisposing factors for cemental tear.^2^ The patient in the above case report did not have a history of traumatic injury or sign of occlusal trauma, thus the possible causative factor was supposed to be that the cementum of this patient was unable to tolerate repeated normal occlusal loading. In addition, the cracks in this patient occurred along the lamellar structure in the thickened cellular cementum, which is the secondary cementum. It was considered the outcome of malnutrition caused by aplastic anemia impairing the function of cementoblasts to secrete intrinsic fibers, thus developing cementum with structural weakness during the active phase of the disease. Therefore, systemic conditions such as malnutrition and aplastic anemia have been speculated to be associated with cemental tear, especially for cases with multiple affected teeth.

### Tooth type

Previous studies suggested that cemental tear largely occurred in single-rooted teeth, especially incisors.^1,7^ However, a recent retrospective study reported that cemental tear was more prevalent in molars, particularly the palatal roots of maxillary molars and the mesial roots of mandibular molars.^32^ The application of CBCT in the latter research might contribute to the difference because CBCT has a more comprehensive view of the affected teeth than conventional X-ray images. Cemental tear often occurs in the middle and apical third of the root.^4,32^ Specifically, it is more commonly found in the middle third of the root of maxillary incisors and the apical third of the root of mandibular incisors or premolars.^32^

### Occlusal trauma or excessive occlusal force

Occlusal trauma is a potential contributing factor to cemental tear and can be classified as primary and secondary trauma.^3,33^ Primary occlusal trauma refers to the damage caused by excess or abnormal occlusal forces acting on healthy periodontium, including bruxism, premature contacts, presence of prosthesis on the antagonist, severe occlusal tooth wear, and abutment teeth of a prosthesis.^3,33^ Secondary trauma occurs when normal or excessive forces are applied to teeth with inadequate periodontal support, typically in periodontitis patients, where attachment loss leads to elevated stress concentration and may result in tooth displacement and increased mobility.^33^ An in vitro study has demonstrated that cementum cracks developed in the weak and stress-concentrated areas such as the cervix of the teeth with repeated or strong stress from the occlusal trauma, and the lesion would progress to the apex with the development of the disease.^24^

### History of periodontitis and periodontal therapy

Periodontal diseases are highly prevalent in developed and developing countries, affecting about 20%-50% of the world’s population.^34^ Pathological alterations in cementum structure are frequently observed in periodontitis patients. Bacterial infiltration and colonization within these pathological niches induce cementum fragmentation and the formation of necrotic cementum fragments.^35,36^ Additionally, it has been demonstrated via scanning electron microscopy that the cementum sections in periodontitis exhibited decreases in the cementum thickness, mineralization, and degradation of collagen fibers.^17^ These structural deteriorations are mechanistically linked to prolonged exposure of cementum to proteolytic enzymes (including host-derived collagenase and matrix metalloproteinases) combined with bacterial metabolic byproducts within the inflammatory microenvironment of periodontal pockets.^17,37^ Root surfaces of teeth extracted from aggressive periodontitis patients are hypoplastic or aplastic, indicating malfunction of cementum deposition.^38^ In addition, the cementum is very thin and less mineralized, which can be damaged or removed easily during mechanical treatment.^35^

### History of dental trauma

Although dental trauma has been revealed not to be a significant risk factor for cemental tear in Lin’s study,^7^ excessive forces induced by trauma are sufficient to separate the cementum from the root surface.^39^ It has been reported that 9.5%–10% of cemetal tear cases have a history of dental trauma.^3,7^ Regarding dental trauma, it is worthwhile to clarify a possible modality, which is the direct damage to the periodontal tissues when overloaded forces are placed on teeth as an anchorage or fulcrum in the process of adjacent teeth extraction.^3,39^ In addition, a case of typical cemental tear occurring in an autotransplanted tooth has also been reported, suggesting a possible association with previous extraction procedures.^40^ Traumatic injury might be a possible predisposing factor for cemental tear, which should be avoided in clinical practice, especially accidental damage to neighboring teeth.

## Clinical manifestations of cemental tear

Clinically, the cemental tear typically presents as hard tissue fragments either completely or partially detached from the root surface, appearing as sheet-like, thin, prickle-like, or tear-like pieces.^3^ Although some cases may be asymptomatic and discovered incidentally, they are more commonly associated with periodontal and/or periapical tissue destruction, as well as signs of occlusal trauma. These lesions are frequently misdiagnosed or inappropriately treated due to their nonspecific presentation. Common clinical signs include gingival or mucosal swelling, isolated deep periodontal pockets^26,41–45^ (Fig. S2a, b), sinus tract or periodontal suppuration,^26,46^ and often a poor response to conventional root canal treatment (RCT).^47^ In cases with periodontal involvement, exploring along the root surface may reveal a hard, ledge-like projection suggestive of a cemental tear, particularly when the lesion communicates with the oral cavity. Additional findings may include bleeding or suppuration on probing, localized rapid attachment loss, increased tooth mobility, and distinct signs of occlusal trauma such as fremitus, wear facets, or premature contacts^48–51^ (Fig. S2d, e). Cemental tears may occur in multiple teeth, and in some cases, may recur on the same tooth.^2^

## Radiographical manifestations of cemental tear

**Fig. 1:**
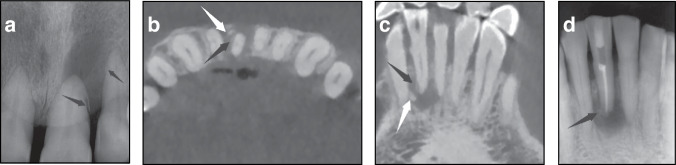
Characteristic radiographic images of teeth with cemental tear. **a** Periapical radiographs of 21 and 22 presented the “prickle-like” radiopaque masses detached from the root surface (black arrows). **b–d** CBCT and periapical radiographs of tooth 41 with cemental tear. Reconstructed CBCT images of 41 in **b** coronal and **c** sagittal views, the black arrow indicating the “flake-like” radiopaque mass detached from the disto-lateral aspect of the root with periapical radiolucent lesions, and the white arrow indicating normal alveolar bone. However, periapical radiograph of 41 **d** after RCT presented with an irregular radiopaque mass (black arrow), which was blurry and easily overlooked, near the disto-lateral aspect at the apical third of the root and periapical radiolucent lesions

Radiographic assessment is an essential procedure for diagnosis, differential diagnosis, treatment, and prognosis evaluation of cemental tear. The typical X-ray appearance of cemental tear is “prickle-like” or “flake-like” (i.e., fine, sharp, and vertical fragments) radiopaque mass near the affected root surface (Figs. S2c and 1a). The radiolucent lesion indicates surrounding alveolar bone resorption accompanied by cemental tear (Figs. S2c and 1a). The radiolucent lesions associated with cemental tear can involve the alveolar crest and apex, manifesting as various patterns, among which periodontal and periapical lesions have been reported in approximately 86% and 65% of the cases, respectively.^7^ Traditional X-rays have limitations in observing 3D objects because biplanar imaging techniques cannot detect cemental tear on the labial (buccal) or lingual (palatal) surface of the teeth.^44,52^ The emergence of CBCT imaging can bridge the gap. When a cemental tear is suspected, small field-of-view CBCT is the preferred examination method, as it can improve the detection rate of cemental tear and disclose their position and extent from a 3D perspective^2,53^ (Fig. 1b–d). In addition, the associated radiolucent lesion can be measured comprehensively.^3,8,54^

## Histopathological manifestations of cemental tear

Histopathological examination of the suspected fragments removed during the scaling and root planing or surgical procedure is regarded as the gold standard for the diagnosis of cemental tear (Fig. S2f, j, m). Histologically, cemental fragments contain cellular and/or acellular cementum, characterized by the presence or absence of cementocytes in lacunae. Torn fragments were often detached at CDJ (77.6%) or within the cementum (22.4%).^1^ Occasionally, torn fragments may include portions of dentin, termed cemento-dentinal tear,^49^ causing more severe dental tissue damage and resulting in non-retainable teeth if too much dentin involvement.^47,55^ Some detached cemental fragments demonstrate obvious periodontal ligament attachment (Fig. S2m). Torn fragments are often partially or completely surrounded by soft tissues, of which 92.3% was granulation tissue, and only 7.7% was cystic.^1^ Many cases involve bone destruction, which is focal destruction of the lamina dura and surrounding medullary bone. The medullary spaces are mostly filled with granulation tissue and/or relatively loose fibrous tissue with lots of chronic inflammatory cells.^5^ The exploration of cemental tear fragments from the destructive periodontal tissues and the histopathological examination are important for the definitive diagnosis.

## Diagnosis of cemental tear

Cemental tear lacks specific clinical signs and symptoms, making diagnosis particularly challenging. In the early stages, patients are often asymptomatic, and the condition may go unnoticed. As the disease progresses, clinical manifestations such as localized periodontal destruction and symptoms resembling pulpitis or apical periodontitis may develop, leading to potential misdiagnosis as periodontal or endodontic disease. Accurate identification requires a thorough diagnostic approach (Table S2), including detailed medical and dental history collection, comprehensive clinical examination—especially probing for hard, step-like projections on the root surface—and radiographic assessment. CBCT is considered particularly valuable in the detection of detached cemental fragments. Ultimately, histopathological examination of the retrieved fragments remains the gold standard for definitive diagnosis.

### Medical and dental history collection

Comprehensive medical and dental history collection is a critical first step in the evaluation of a suspected cemental tear. Clinicians should begin by assessing the patient’s general systemic health and chief dental complaints. If a cemental tear is suspected, further detailed inquiry should be conducted regarding potential predisposing factors. These include the presence of parafunctional habits such as bruxism or clenching, frequent consumption of hard foods, pain during mastication, bleeding during tooth brushing, and any history of periodontal or root canal treatment. Special attention should also be given to any history of dental trauma or prosthetic procedures. Such information is essential for identifying risk factors associated with cemental tear and provides valuable clues for its differential diagnosis from other clinically similar conditions.

### Clinical examination

#### Occlusal examination

Determination of traumatic occlusion through occlusal analysis, including visual inspection, palpation technique, and use of articulating paper. Referral to specialists should be considered without hesitation whenever appropriate.

- **Visual examination:** The first step for an occlusal assessment, including tooth positioning and alignment (occlusal relationship), wear patterns on teeth, etc., provides preliminary insights into the occlusal landscape of the patient, followed by further examination.
- **Palpation technique:** Determination of fremitus, vibration, or micromovement of a tooth through lightly pressing the finger on the labial (buccal) surface of the maxillary teeth while patients tap their teeth simultaneously or mimic clenching and attempt to move the mandible in excursions. Teeth with greater vibration or mobility may have premature contact.
- **Use of articulating paper:** The occlusal contacts can be identified by placing ultra-thin articulating papers between the upper and lower teeth and instructing the patient to bite in centric occlusion, excursion laterally, and protrusion forward. Red and blue articulating papers indicate the occlusal contacts of static and dynamic occlusion, respectively. The location and range of occlusal contacts can be known by revealing the red and blue markings on the tooth surface. Based on these examination results, premature contacts and occlusal interference areas will be adjusted. In complicated scenarios, further examination by a specialist may be required.

#### Periodontal condition

Oral hygiene status, location of the gingival margin, and gingival condition through visual inspection; root surface irregularities, bleeding or suppuration on probing, probing depth (PD), and periodontal attachment loss; tooth mobility by applying pressure in a facial-lingual direction as well as in a vertical direction. Detection of root surface irregularities is important for the suspected diagnosis of cemental tear, as the torn fragments may feel rough, jagged, or uneven when explored using a sharp-ended dental explorer along the root surface. In addition, periodontal endoscopy is preferred to disclose the partially or completely detached fragments.

#### Tooth hard tissues, pulp, and periapical condition

Disclosure of sinus tract, tooth wear, caries, defects, restorations, and tooth crack through visual inspection; evaluation of pulp status by cold, heat, and electric pulp tests; determination of periapical abnormalities through vertical percussion. For the tooth with a sinus tract, clinicians should suspect the presence of a cemental tear if no endodontic diseases or failed endodontic treatment are present, especially for teeth with vital pulp.

### Auxiliary examination

#### Radiographic examination

Detection of a “prickle-like” or “flake-like” radiopaque mass near the root surface of the affected tooth is usually the critical evidence for the diagnosis of cemental tear. The differential diagnosis of the “prickle-like” radiopacity includes lamina dura and residual alveolar bone. In cemental tear, however, these radiopaque fragments typically appear attached to the root surface or separate from it and are located within a radiolucent lesion.^2^ CBCT is preferred over traditional X-rays for better detecting cemental tear, especially on labial (buccal) or lingual (palatal) surfaces, and for detailed evaluation of associated radiolucent osseous lesions.

#### Methylene blue dye staining

Methylene blue dye staining can help clinicians identify the boundary of cemental tear and detect otherwise invisible cracks during the periodontal or periapical surgery and intentional replantation, which is conducive to the complete removal of torn fragments.^55,56^

#### Histopathological examination

Histopathological examination remains the gold standard for the definitive diagnosis of cemental tear.^3,55^ In clinical practice, detached cemental fragments may be obtained during scaling, root planing, periodontal or periapical surgery for histopathological analysis. However, in certain cases, exploratory surgery is required to retrieve these fragments, which may pose a risk of postoperative complications. Therefore, clinicians should carefully weigh the benefits and risks of exploratory procedures as part of the diagnostic strategy. In cases where the affected tooth has a hopeless prognosis, unnecessary surgical intervention should be avoided to minimize patient burden and psychological distress.^3^ It is recommended that all removed fragments be examined histopathologically to confirm the diagnosis and guide subsequent treatment.

## Differential diagnosis of cemental tear

Most clinical features associated with cemental tear are also common in other pathological conditions involving periodontal and periapical lesions, such as VRF, primary endodontic diseases, and primary periodontal diseases^8,39^ (Table S3). Since the clinical management and prognosis of different diseases vary, careful differential diagnosis is pivotal for clinicians to provide appropriate treatment plans in cases with periodontal and endodontic lesions. In this section, we briefly introduce the differential diagnosis of cemental tear. Radiographic and histopathological examinations remain essential tools for preliminary and definitive diagnosis, respectively.

### VRF

Tooth extraction is often the most predictable treatment for teeth diagnosed with VRF due to their poor prognosis. In contrast, teeth affected by cemental tear usually remain functional after appropriate treatment.^57^ Therefore, careful examination of teeth with isolated periodontal pockets and narrow bone defects is crucial to avoid unnecessary extractions. VRF is more commonly associated with root filled teeth,^57^ while cemental tear itself usually has no influence on pulp tissue thus the affected teeth are mainly with vital pulp if no previous RCT or apical involvement.^3,7,58^ Radiographic examination is the key tool for differential diagnosis. In cemental tear, thin radiopaque fragments can be seen along the root surface. In contrast, VRF typically presents as a vertical separation of the root structure, with a radiolucent line between the root filling material and the root surface.^57,59^

### Primary endodontic diseases

Sinus tract is the common clinical feature of both cemental tear and primary endodontic diseases. However, the pulp can remain vital even in the presence of a sinus tract in cases of cemental tear.^48,60^ For teeth without previous RCT, differential diagnosis is easier because problems of endodontic origin usually show inflammatory or necrotic pulp and careful evaluation of pulp vitality is important to avoid performing RCT in vain. In addition, identification of “flake-like” radiopaque mass separated from the root surface via radiographic examination is always useful information for differential diagnosis of cemental tear and primary endodontic diseases, especially for teeth with previous adequate RCT but no relief.^8,26,61^

### Periodontal diseases

Deep periodontal pocket, periodontal suppuration, and bleeding on probing are common clinical features of both cemental tear and periodontal diseases. Overlooking of cemental tear would hinder the healing of periodontal tissues.^60,62,63^ Therefore, careful inspection of radiographic images is necessary to clarify the primary cause of periodontal lesions (Fig. S2). In addition, periodontal diseases usually involve multiple teeth and induce generalized bone loss instead of localized lesions associated with specific root surfaces. Although periodontal disease can also present in the form of localized abscesses occasionally, the detection of ledge-like projections on the root surface could facilitate distinguishing cemental tear from periodontal abscess. The typical radiographic and histological manifestations of cemental tear are always critical features for differential diagnosis.

## Clinical management of cemental tear

**Fig. 2:**
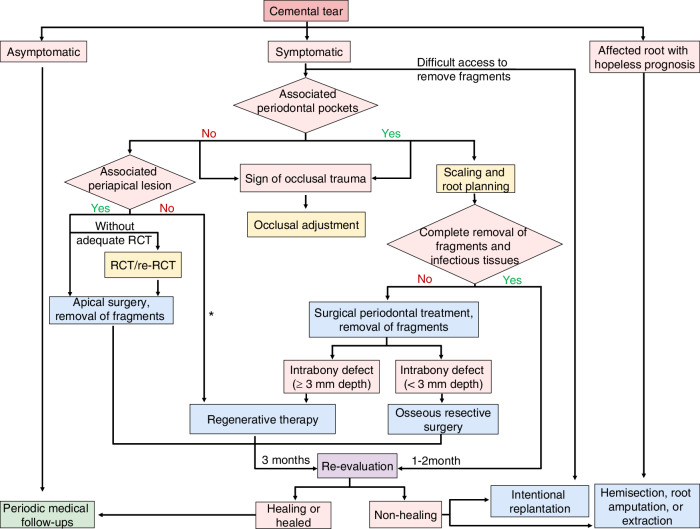
Decision tree of treatment plans for cemental tear. (*RCT* root canal treatment, *re-RCT* root canal retreatment, the asterisk (*) denotes that neither fragments nor associated bone defects involve the alveolar crestal bone and the apex of the root)

The principal objectives of cemental tear treatment are to thoroughly remove torn fragments and infected tissues and reconstruct the structure and function of the affected tissues. Current treatment modalities for cemental tear mainly include periodic medical follow-ups, occlusal adjustment, scaling and root planning, RCT, surgical treatment, and tooth extraction (Table S4). The treatment strategy for cemental tear is primarily based on the location of the torn fragment, the severity of associated periodontal and periapical lesions, and the patient’s preference.^3,4^ Thus, we proposed a treatment decision tree mainly based on the condition of periodontal and periapical lesions and accessibility of torn fragments to guide clinicians in selecting appropriate interventions (Fig. 2). For cemental tear in the cervical third of the root, complete removal of torn fragments and infected tissues can sometimes be achieved through scaling and root planning alone. If there are residual fragments and infectious tissues, surgical treatments are required, usually including periodontal surgery, apical surgery, regenerative therapy, intentional replantation, hemisection, and root amputation. Lee et al. proposed a classification of cemental tear and corresponding treatment strategies recently^3^; however, large-scale studies and evidence on the treatment of cemental tear are currently limited. Further clinical research is needed to provide more comprehensive diagnostic and therapeutic strategies.

## Current treatment modalities of cemental tear

### Periodic medical follow-ups

No active intervention is necessary for patients with only radiographic findings but with no clinical signs and symptoms.^25^ However, it is important to inform the patient and schedule regular follow-ups. Intervention treatment should be implemented if any sign of disease progression appears.^3,49^

### Occlusal adjustment

Occlusal adjustment is necessary when the affected tooth has traumatic occlusion or pronounced looseness. The presence of these problems may interfere with occlusal stability and lead to occlusal discomfort during functional tooth movement, potentially exacerbating the progression of cemental tear and associated lesions, as well as affecting the healing of periodontal lesions.^64,65^ Occlusal adjustment has been demonstrated to be conducive to obtaining the clinical periodontal attachment gain after periodontal treatment.^66–68^ Therefore, occlusal examination and adjustment are important parts of the whole therapeutic process. Harmonious occlusal function is beneficial for tissue healing. Thus, necessary tooth splinting for stabilizing mobile teeth should be considered before any surgical intervention and regenerative periodontal therapy.^69^

### Subgingival scaling and root planning

Subgingival scaling and root planning can remove the torn cementum that is completely reachable through the periodontal pockets. Sometimes, the completely separated cemental fragment which was projecting into periodontal pocket can be removed with the aid of locking pliers.^25^ In addition, performing subgingival scaling and root planning with a dental operating microscope, such as a periodontal endoscope, is indispensable to improve visualization and facilitate the complete removal of cemental tear and infected granular tissues.^3^ However, only 28.6% of cases were cured after the non-surgical periodontal treatment, as the periodontal pocket approach provides limited visualization and narrow operating space, potentially leaving some torn fragments and infected tissues, causing delayed healing or non-healing.^3,70^ Therefore, periodontal re-evaluation is essential for patients with cemental tears that are removed through non-surgical treatment after 1–2 months.^71^ Further surgical periodontal therapy should be considered if there is no improvement in the periodontal condition.^48,72,73^

### RCT

The presence of cemental tear is not thought to affect pulp vitality. RCT is unnecessary for teeth without the involvement of the root apex, and the pulp vitality needs to be carefully tested. However, cemental tear in the apical third of the root usually accompanies periapical lesions, which may lead to pulp necrosis when the periapical lesions communicate with the oral cavity. In addition, apical resection as a step of the apical surgery procedure may undermine the apical neurovascular supply to the pulp.^74,75^ For the above situations, adequate RCT is required.^3^

### Surgical therapy

#### Periodontal surgery

Surgical intervention is required when a cemental tear occurs in the middle third and apical third of the root, or the deep periodontal pocket (PD > 5 mm) persists after non-surgical periodontal treatment.^42,45^ Through surgical treatment, 57.7% of cases healed, including the repair of surrounding bone defects.^70^ To thoroughly remove cemental fragments and reduce postoperative consequences, microscopes and microsurgical instruments can be employed.^59,76^ Debridement is necessary. In addition, the involved root surface can be smoothed using diamond-coated burs.^77^ Cemental tear is often accompanied by intrabony defects (vertical bone defects). Thus, regenerative surgery should be considered based on the systemic condition of patients, intrabony defects evaluation (e.g., vertical depth, defect angle or width, number of bony walls, and esthetic factor), and patient’s desire.^78^ If the affected tooth is suitable for surgery, the choice of a regenerative approach is generally based on the analysis of intrabony defect features.^78^ Firstly, the decision to regenerate depends on the vertical depth of the intrabony defects. Bone regeneration is difficult for early or shallow intrabony defects (<3 mm depth), while osseous resective surgery is suitable. For deep intrabony defects (≥3 mm depth), regenerative surgery using enamel matrix derivative (EMD) or guided tissue regeneration (GTR) shows significant periodontal improvement compared with open flap surgery.^79–81^ Then, the decision to use regenerative materials depends on the width of intrabony defects and the number of bony walls. For narrow 3-wall intrabony defects (<2 mm width), bone grafts, GTR, or biological factors can be applied in regenerative procedures separately. For wide 3-wall intrabony defects (≥3 mm width) and two-wall intrabony defects, bone grafts should be combined with GTR or biological factors (such as EMD). For 1-wall intrabony defects, the combination of bone grafts, biological factors, and GTR should be utilized.^78^ Among intrabony defects with different walls, narrow two-wall or three-wall intrabony defects show the most significant potential for regeneration.^78,82^ It should be noted that esthetic risk assessment should be conducted for cemental tear occurring in anterior teeth.

#### Apical surgery

Minimally invasive apical surgery is required to completely remove the fragments and infectious tissues at the apical region of the root.^3,62^ In addition, regenerative therapy could be a favorable option if the diameter of the periapical bone defect is ≥10 mm or the height of the residual buccal bone plate is ≤3 mm.^2,3,42,83,84^ For periapical defects extending from the buccal to the lingual bone plate, combined approach using bone grafts and regenerative membrane shows favorable prognosis.^84^ Apico-marginal lesion is the most challenging situation in apical surgery. Clinicians should be cautious to perform apical surgery when the buccal (and/or mesial-distal) root surface is completely exposed, although regenerative barrier membranes yield better results.^84^

#### Intentional replantation, hemisection, and root amputation

When the affected tooth is near important vascular and neural anatomical structures and/or when access to completely remove the torn fragment is difficult, intentional replantation is a feasible option,^85,86^ even in the process of periodontal open flap surgery.^87^ Hemisection or root amputation might be valid treatment plans for affected teeth with multiple roots.^4,88^

### Tooth extraction

For teeth with poor or hopeless prognosis, such as severe bone resorption and extensive cemental tear involvement, or when the patient has a strong preference for extraction, early extraction can be considered to avoid further bone destruction,^89,90^ since local soft and hard tissues will deteriorate after tooth loss. Therefore, alveolar ridge preservation or immediate implantation is a feasible option to preserve soft and hard tissues, thus facilitating subsequent restoration and improving restoration quality.^91,92^ Additionally, traumatic damage during the extraction process may exacerbate postoperative bone resorption, so minimally invasive extraction techniques should be employed to avoid pressure on thin labial/lingual bone.^92^

## Treatment outcome of cemental tear

Complete removal of torn fragments is crucial to treatment outcomes.^3,55^ It has been reported that 94% of treated teeth after cemental tear removal were functionally retained after 1-year follow-up; thus, active intervention of cemental tear is recommended.^70^ The treatment outcome of the cemental tear depends on the coronal-apical location of the torn fragments, with healing rates of 60%, 66.7%, and 11.1% for tears in the coronal third, middle third, and apical third of the root, respectively.^70^ In addition, a higher healing rate was demonstrated in affected teeth with surgical intervention compared with non-surgical treatment alone.^70^

## Conclusion

**Fig. 3:**
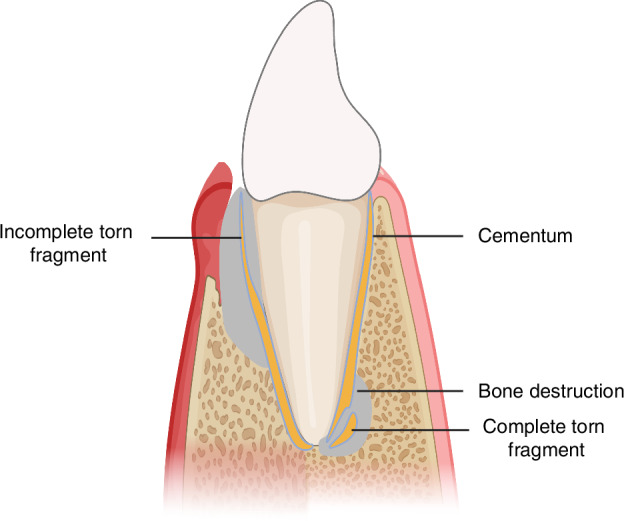
Schematic image of cemental tear

**Fig. 4:**
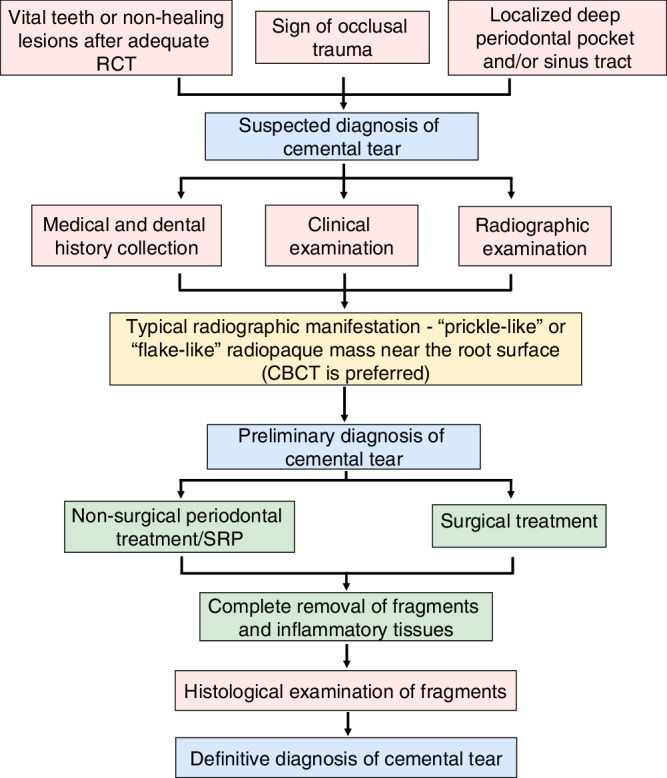
Summary figure of diagnosis and treatment procedure of cemental tear

The cemental tear is illustrated by a partial or complete detachment of the cementum from the CDJ or along the incremental line within the body of the cementum and, in some cases, with adjacent bone resorption around the affected root (Fig. 3). The potential mechanism of cemental tear formation can be divided into internal factors and external factors. The former includes the weakness of CDJ and cementum, and the latter involves stress applied to the cementum, like occlusal trauma. Age, gender, tooth type, occlusal trauma, history of periodontitis and periodontal treatment, and history of dental trauma compose possible predisposing factors (Table S1). The preliminary diagnosis of cemental tear mainly relies on radiographic examination, and CBCT is the preferred choice (Table S2, Fig. 4). For the treatment of cemental tear, complete removal of torn fragments is essential for tissue healing (Fig. 4), and regenerative therapy is favorable for suitable cases (Table S4). In conclusion, the successful treatment of cemental tears is feasible if correctly diagnosed, and referral to endodontists and/or periodontal specialists is highly recommended if necessary.

## Supplementary information

Supplemental Material
