# Supplementary material for: Expert consensus on the diagnosis and treatment of cemental tear
Source: Int J Oral Sci. 2025 Aug 22;17:61. doi: 10.1038/s41368-025-00381-9 (PMC12373928; doi:10.1038/s41368-025-00381-9)
Supplement: Supplementary file 1 — Supplemental Material [file 41368_2025_381_MOESM1_ESM.docx]

Supporting Information

Supplementary Figures


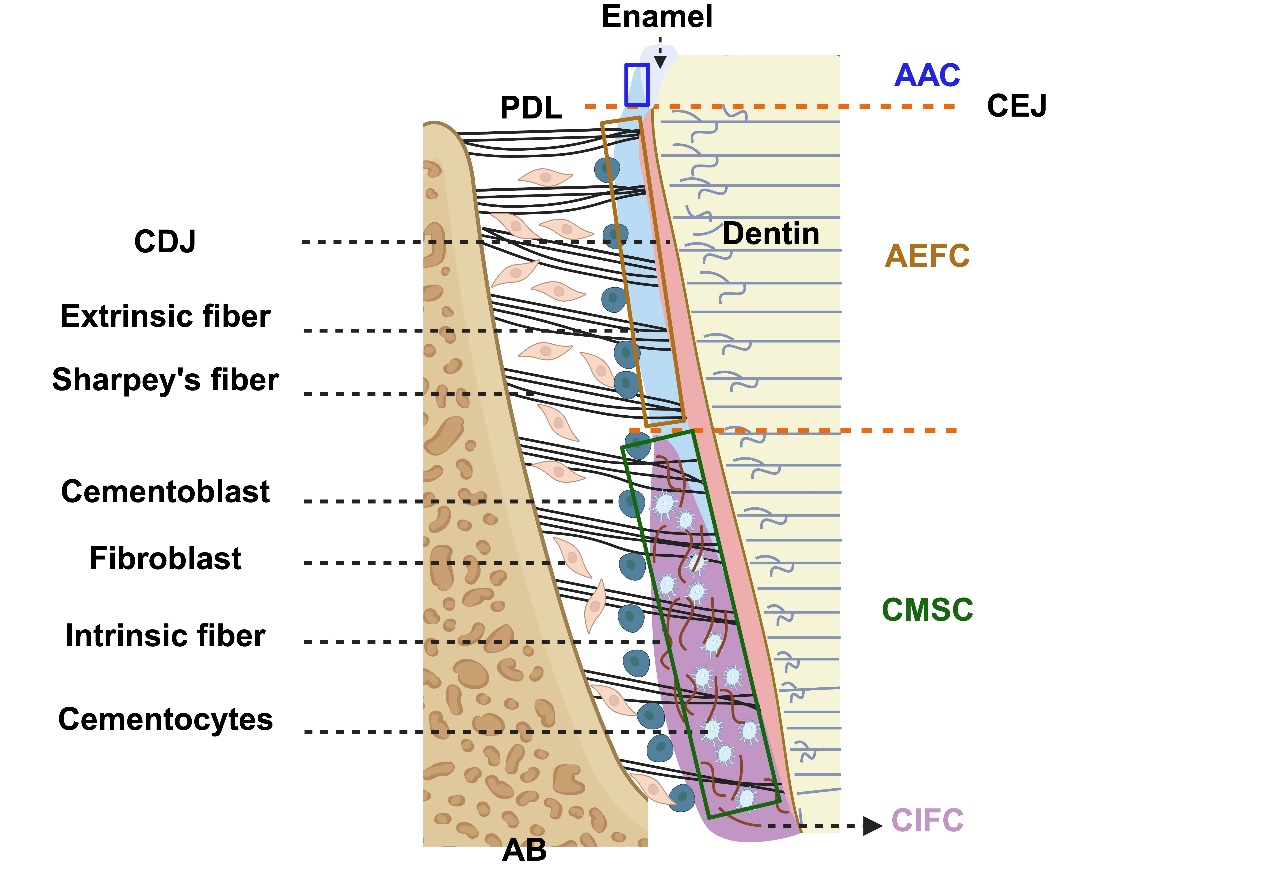


**Fig. S1** Schematic illustration of cementum. (Notes: AB=alveolar bone, PDL=periodontal ligament, CDJ=cemento-dentinal junction, CIFC=cellular intrinsic fiber cementum, AAC=acellular afibrillar cementum, AEFC=acellular extrinsic fiber cementum, CMSC=cellular mixed stratified cementum，CEJ=cemento-enamel junction)


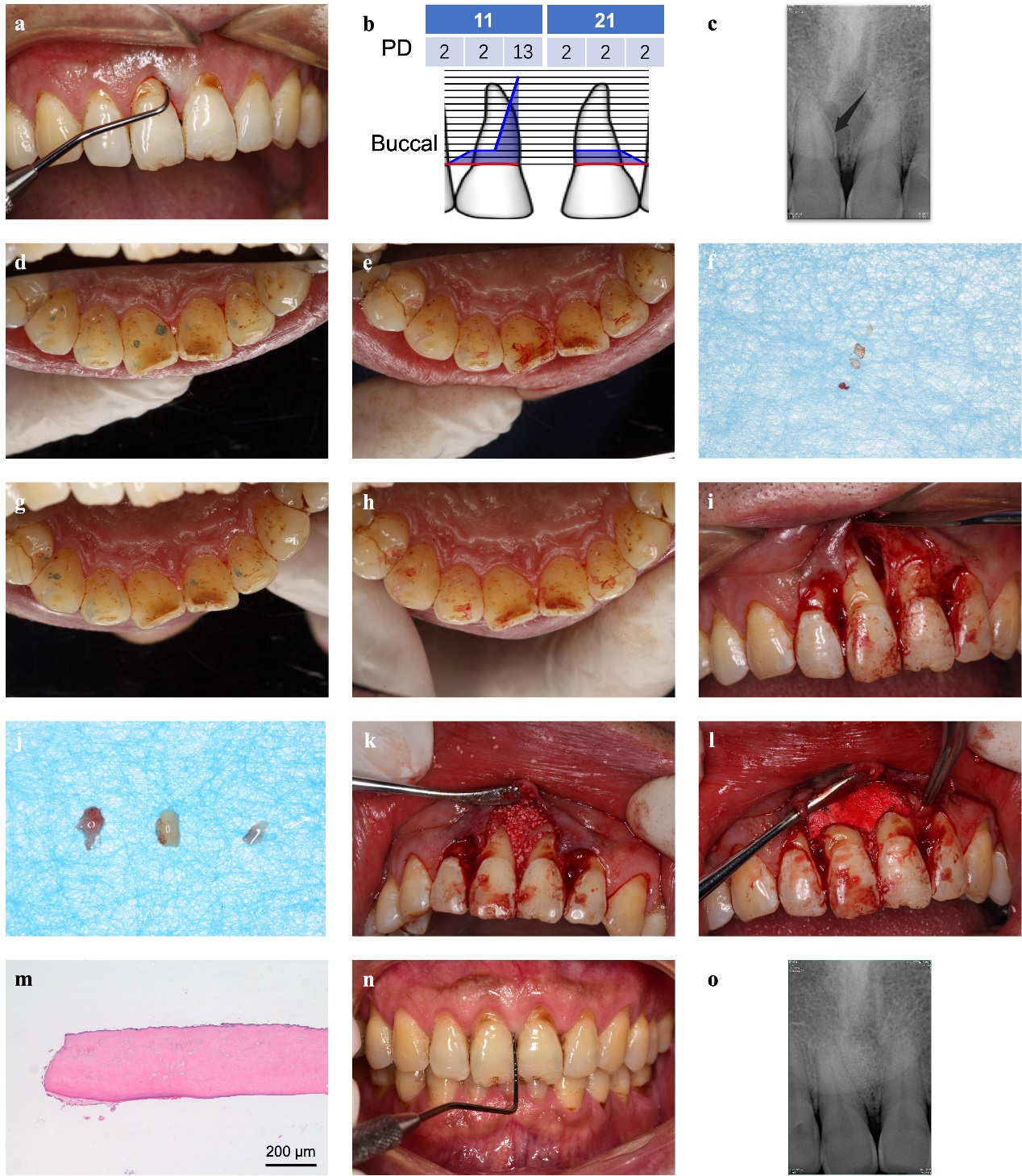


**Fig. S2** Example of diagnosis and treatment procedure of tooth with cemental tear^50^. A 62-year-old male came for treatment with the chief complaint of "repeated swelling and purulent of gingiva in the right upper anterior for 3 years". Previous periodontal treatment at another hospital with no relief. With hard food eating habit. Pre-operative clinical view of tooth 11 (**a**) and probing depth (PD) of teeth 11, 21 (**b**) presented the isolated deep periodontal pocket. **c** Pre-operative periapical radiograph of tooth 11 presenting with “prickle-like” radiopaque mass (black arrow) at the mesal aspect involving cervical 2/3 part of the root indicating the suspected diagnosis of cemental tear and associated radiolucency without apical involvement. **d, e** Pre-operative clinical view of occlusal examination with blue dots showing the occlusal contacts of static occlusion and red dots showing the occlusal contacts in protrusive occlusion, indicating the presence of occlusal trauma on tooth 11. **f** Several fragments were removed during scaling and root planning, but there were residual fragments because of the limited accessibility of non-surgical periodontal treatment for the deep periodontal pocket. **g, h** Clinical view of occlusal examination after occlusal adjustment. **i** Periodontal surgery was further performed to remove the residual fragments and regenerate periodontal tissue, and 2-wall intrabony defect without palatal plate involvement was exposed after the open flap surgery. **j** Several pieces of residual fragments were further removed. **k, l** The xenobone graft material (Bio-oss, Geistlich, Sweden) and resorbable collagen membrane (Oral Cavity Repair Membrane Type C, Heal-All, China) were utilized for the regenerative therapy. **m** Hematoxylin and Eosin staining image showed acellular cementum with adjacent periodontal ligament. **n, o** At the 1-year postoperative visit, the clinical view of tooth 11 showed healthy gingiva with PD of less than 3 mm and the periapical radiograph indicated no abnormal findings. Adapted from Shao et al. (2022), Chinese Journal of Stomatology, 57(8), p. 871-873. Copyright 2022 by Chinese Medical Association. Reprinted with permission.

Supplementary Tables

**Table S1.** Possible predisposing factors of cemental tear

| Possible predisposing factors | Further information and illustrations |
| --- | --- |
| Age | Mainly over 60 years old^3,7^. |
| Gender | Mainly in male^3,4,7^. |
| Tooth type | Controversial, mainly incisors in previous studies^1,7^, but mostly molars (71.43%) in a recent study^32^. |
| Systemic condition | Malnutrition and aplastic anemia might affect the function of cementoblast forming secondary cementum with structural weakness^2^. |
| Occlusal trauma | Primary occlusal trauma and secondary occlusal trauma^3,33^. |
| History of periodontitis | Pathogenic changes of cementum in inflammatory situation^17^. |
| History of dental trauma | 9.5-10% of the cases presented with a history of dental trauma^3,7^. |

**References:**

1 Lin, H. J. *et al.* Clinical fracture site, morphologic and histopathologic characteristics of cemental tear: role in endodontic lesions. *J Endod* **38**, 1058-1062, doi:10.1016/j.joen.2012.04.011 (2012).

2 Watanabe, C., Watanabe, Y., Miyauchi, M., Fujita, M. & Watanabe, Y. Multiple cemental tears. *Oral Surg Oral Med Oral Pathol Oral Radiol* **114**, 365-372, doi:10.1016/j.oooo.2012.01.003 (2012).

3 Lee, A. H. C., Neelakantan, P., Dummer, P. M. H. & Zhang, C. Cemental tear: Literature review, proposed classification and recommendations for treatment. *Int Endod J* **54**, 2044-2073, doi:10.1111/iej.13611 (2021).

4 Jeng, P. Y. *et al.* Cemental tear: To know what we have neglected in dental practice. *J Formos Med Assoc* **117**, 261-267, doi:10.1016/j.jfma.2017.09.001 (2018).

7 Lin, H. J. *et al.* Cemental tear: clinical characteristics and its predisposing factors. *J Endod* **37**, 611-618, doi:10.1016/j.joen.2011.02.017 (2011).

17 Parlak, H. M. *et al.* Analysis of the nano and microstructures of the cervical cementum and saliva in periodontitis: A pilot study. *J Oral Biosci* **63**, 370-377, doi:10.1016/j.job.2021.09.007 (2021).

32 Zhao, S., Yuan, Z., Zhou, X. & Yang, X. Clinical, radiographic features and prognosis of cemental tear: A retrospective study of 63 teeth. *Heliyon* **10**, e30999, doi:10.1016/j.heliyon.2024.e30999 (2024).

33 1999 International International Workshop for a Classification of Periodontal Diseases and Conditions. Papers. Oak Brook, Illinois, October 30-November 2, 1999. *Ann Periodontol* **4**, i, 1-112, doi:10.1902/annals.1999.4.1.i (1999).

**Table S2.** Diagnosis of cemental tear

| Diagnosis procedures | Key points |
| --- | --- |
| Medical and Dental History Collection | Oral parafunction, such as bruxism or tooth clenching; favoritism for eating hard food. |
| Clinical Examination | |
| *Periodontal condition* | Deep isolated periodontal pocket; bleeding or suppuration on probing; detection of root surface irregularities; periodontal endoscopy is favorable for disclosing the fragments. |
| *Tooth hard tissues, pulp, and periapical condition* | Sinus tract; tooth wear; mainly vital pulp. |
| *Occlusal examination* | Occlusal trauma. |
| Auxiliary Examination |  |
| *Radiographic examination* | The critical evidence for the diagnosis of cemental tear: “prickle-like” or “flake-like” radiopaque mass near the root surface; small field-of-view CBCT is preferred. |
| *Methylene blue dye staining* | Identifying the boundary of cemental tear during surgical treatment. |
| *Histopathological examination* | Gold standard for the diagnosis of cemental tear: detached cellular and/or acellular cementum. |

**Table S3.** Differential diagnosis of cemental tear

| Condition | Cemental tear | VRF | Primary endodontic disease | Periodontal disease |
| --- | --- | --- | --- | --- |
| Definition and key features | Partially or completely detachment of cementum from the root surface at CDJ or within the body of cementum along the incremental lines.  Histopathological examination of the detached fragment indicates cellular and/or acellular cementum. | Fractures along vertical axis of the root, expanding from the root canal wall laterally to the root surface in a horizontal aspect.  Definitive diagnosis of VRF is best determined by exploratory surgery. | Infection originating from pulp, may present with a sinus tract or swelling.  The existing problem of endodontic origin, like caries. | Inflammatory destructive disease of periodontal tissue.  Loss of soft tissue attachment and alveolar bone resorption, swelling, redness, bleeding on probing, and purulence. |
| Pulpal status | Vital (unless with previous RCT or surrounding bone lesions involving apex to cause pulpal necrosis).  The location of sinus tract is associated with the position of torn fragment. | Usually in teeth with previous RCT.  The location of sinus tract is close to the gingival margin. | Pulpal necrosis/non-vital.  Responding well to RCT. | No effect on the pulp viability, unless the periodontal destruction involves the apex or furcation area. |
| Characteristics of periodontal condition | Deep periodontal pocket localized to the specific root area.  Irregular root surface with a hard and ledge-like projection. | One or two narrow deep periodontal pockets (especially at both buccal and lingual aspects) localized to the fracture site. | No deep periodontal pocket formation unless periapical lesions destroy the integrity of periodontal tissue. | Deep periodontal pockets with generalized bone loss, and multiple teeth involvement.  Bone loss is usually a slow process, except periodontal abscess. |
| Radiographic findings | The radiographic images show that "prickle-like" or "flake-like" radiopaque mass (detached fragment) along the root surface, and some with associated radiolucent lesions. | The appearance of hair-like fracture line radiolucency in the dentin body or the radiographic appearance of root segment separation.  “Halo” appearance, a combination of periapical and perilateral radiolucency. | Radiolucency at the apex, not along the root surface. | Multiple teeth with alveolar bone loss (horizontal/vertical), furcation involvment. |

**Table S4.** Treatment modalities of cemental tear

| Treatment modalities | Indications |
| --- | --- |
| Periodic review | Only radiographic findings with no clinical signs and symptoms. |
| Occlusal adjustment | Affected teeth with traumatic occlusion or pronounced looseness. |
| Subgingival scaling and root planning | The torn cementum is completely removable through the periodontal pockets. |
| RCT | Affected teeth with necrotic pulp or scheduled for apical surgery |
| Surgical therapy | |
| *Periodontal Surgery* | The torn cementums in the middle third of the root that are incompletely removable through the periodontal pockets; persistent deep periodontal pocket (PD > 5 mm) after non-surgical periodontal treatment; regenerative therapy is considered if the intrabony defect with over 3 mm depth. |
| *Apical Surgery* | The torn cementum is in the apical third of the root; regenerative therapy is considered if the bone defect has a diameter of over 10 mm. |
| *Intentional Replantation* | Affected teeth near important vascular and neural anatomical structures and/or with difficult access. |
| *Hemisection and Root Amputation* | Affected teeth with multiple roots. |
| Tooth Extraction | Affected teeth with poor or hopeless prognosis. |
